# VTE Risk Assessment Compliance in Older Adult Psychiatric Inpatients

**DOI:** 10.1192/bjo.2026.11733

**Published:** 2026-06-30

**Authors:** Tegan Deu, Aparna Prasanna

**Affiliations:** Black Country Healthcare NHS Foundation Trust, Wolverhampton, United Kingdom

## Abstract

**Aims:**

The aim of this audit is to assess adherence to the local VTE (venous thromboembolism) risk assessment guidelines across inpatient older adult psychiatry care in the Black Country Healthcare NHS Foundation Trust (BCHFT). An update to the National Institute for Health Care and Clinical Excellence (NICE) VTE risk reduction guidelines in 2018 included specific guidance for assessing acute psychiatric patients. In line with this, the BCHFT VTE risk assessment guidelines were updated in 2024. These local guidelines state that patients should be risk assessed within 14 hours of admission, at first multi-disciplinary (MDT) review, and at any change in clinical condition during admission. Additionally, local guidelines include the assessment of psychiatry related risk factors for thrombosis.

**Methods:**

The patient electronic records were reviewed retrospectively for all older adult inpatients (n=58) across the BCHFT trust on the 1st January 2026. All patients had been inpatients for at least one week. Data was collected on the time from admission to first risk assessment, whether risk assessments were updated at MDT review, and whether any risk assessments were updated due to change in clinical condition. Secondly, data was collected on the number of risk assessments that included psychiatric specific risk factors where appropriate.

**Results:**

69% (n=40) of patients had a VTE assessment completed. 55% (n=22) were female and 45% (n=18) were male. 64% (n=37) had their assessment completed within 14 hours of admission. 2% (n=1) of patients had an updated VTE assessment at first MDT review. 9% of patients (n=5) had another VTE assessment completed on re-assessment, due to change in clinical condition. 0% of VTE assessments (n=0) included psychiatric specific risk factors in their assessment, such as anti-psychotic use, severe depression or catatonia.

**Conclusion:**

There is considerable scope for improvement in VTE risk assessment compliance across the BCHFT trust, particularly regarding VTE re-assessment at first MDT review. Additionally, incorporation of psychiatric risk factors in the assessment has not been implemented in practice, despite its inclusion in the local guidelines. Recommendations include updating the electronic patient record templates to be in line with local and national guidelines. Further education for MDT members is planned to ensure the completion of re-assessments within necessary time limits. These results are to be presented at local trust teaching, with the aim to re-audit in one year.

